# Hyaluronic acid-serum albumin conjugate-based nanoparticles for targeted cancer therapy

**DOI:** 10.18632/oncotarget.15363

**Published:** 2017-02-15

**Authors:** Ravit Edelman, Yehuda G. Assaraf, Inna Levitzky, Tal Shahar, Yoav D. Livney

**Affiliations:** ^1^ Department of Biotechnology and Food Engineering, Technion-Israel Institute of Technology, Haifa, 32000, Israel; ^2^ Department of Biology, Technion-Israel Institute of Technology, Haifa, 32000, Israel; ^3^ Russell Berrie Nanotechnology Institute, Technion-Israel Institute of Technology, Haifa, 32000, Israel

**Keywords:** hyaluronic acid, Maillard reaction, nanovehicles, cancer, targeted therapy

## Abstract

Multiple carcinomas including breast, ovarian, colon, lung and stomach cancer, overexpress the hyaluronic acid (HA) receptor, CD44. Overexpression of CD44 contributes to key cancer processes including tumor invasion, metastasis, recurrence, and chemoresistance. Herein, we devised novel targeted nanoparticles (NPs) for delivery of anticancer chemotherapeutics, comprised of self-assembling Maillard reaction-based conjugates of HA and bovine serum albumin (BSA). HA served as the hydrophilic block, and as the ligand for actively targeting cancer cells overexpressing CD44. We demonstrate that Maillard reaction-based covalent conjugates of BSA-HA self-assemble into NPs, which efficiently entrap hydrophobic cytotoxic drugs including paclitaxel and imidazoacridinones. Furthermore, BSA-HA conjugates stabilized paclitaxel and prevented its aggregation and crystallization. The diameter of the NPs was < 15 nm, thus enabling CD44 receptor-mediated endocytosis. These NPs were selectively internalized by ovarian cancer cells overexpressing CD44, but not by cognate cells lacking this HA receptor. Moreover, free HA abolished the endocytosis of drug–loaded BSA-HA conjugates. Consistently, drug-loaded NPs were markedly more cytotoxic to cancer cells overexpressing CD44 than to cells lacking CD44, due to selective internalization, which could be competitively inhibited by excess free HA. Finally, a CD44-targeted antibody which blocks receptor activity, abolished internalization of drug-loaded NPs. In conclusion, a novel cytotoxic drug-loaded nanomedicine platform has been developed, which is based on natural biocompatible biopolymers, capabale of targeting cancer cells with functional surface expression of CD44.

## INTRODUCTION

Active targeting based on molecular recognition is an effective modality to enhance drug selectivity and efficacy, and to reduce chemotherapeutic drug doses and side effects [1–3]. One approach to achieve active targeting is by designing NPs decorated with hyaluronic acid (HA), to target the CD44 receptor, which is overexpressed on the surface of various carcinomas [4, 5]. HA is a glycosaminoglycan of the extracellular matrix consisting of tandem repeats of D-glucuronic acid and *N*-acetyl-D-glucosamine [6]. Since HA is a physiological constituent, it is biocompatible and non-immunogenic and could therefore be an ideal biomaterial for drug delivery and tissue engineering [7]. Many of the downstream pathways following CD44 activation become deregulated in cancer, leading to tumor growth, progression and metastasis [5, 8]. During carcinogenesis, expression of the standard CD44 form is up-regulated in various carcinomas including breast, ovarian, colon, lung and stomach cancer [9, 10]. HA oligosaccharides show high affinity to CD44 receptors [5].

Serum albumin (SA) is an abundant globular protein whose remarkable water solubility is due to the presence of a large number of ionizable amino acid side groups on its surface [11, 12]. The primary physiological function of SA is to transport proteins, metal ions, lipids and other metabolites through the plasma [13]; hence, SA is a highly proficient carrier for drugs, in particular lipophilic drugs [14–16]. Since bovine serum albumin (BSA) is highly homologous to human serum albumin (HSA), we used it here as a model, with the aim to use HSA in future clinical studies. The amine side groups of certain amino acids in SA including lysines, arginines, histidines and tryptophans can react with the reducing aldehyde group of HA by the Maillard reaction [17, 18]. The latter is a non-enzymatic browning reaction, common in food, which is initiated by heating a dry mixture or a solution of a carbonyl-containing compound (usually a reducing sugar) and an amine residue-containing compound, usually an amino acid, a peptide or a protein [17–19]. The Maillard reaction is accelerated by heat, without relying on catalyst molecules, and it is capable of improving the functional properties of the protein such as solubility, heat stability, self-assembly and emulsifying capacity [17, 20]. Maillard reaction-based conjugates of HA and SA have not been reported to date. We hypothesized that Maillard HA-SA conjugates would form a self-assembling, actively targeted, biocompatible and biodegradable polymeric nano-carrier, which would be able to entrap hydrophobic chemotherapeutics. Hence, this will provide major advantages in hydrophobic drug encapsulation, stabilization, selective targeting, and controlled release. HA bound to its CD44 receptor may be actively internalized into cells via receptor-mediated endocytosis, followed by lysosomal proteolytic breakdown and intracellular release of the cytotoxic drug cargo [5, 7]. Thus, HA may simultaneously serve as both the hydrophilic external domain of the block-copolymeric nano-carrier and as the targeting ligand, to achieve a Trojan horse effect [7, 21].

Paclitaxel (PTX), a hydrophobic chemotherapeutic drug that is widely used against a spectrum of solid tumors [22, 23], blocks mitosis by binding to β-tubulin, resulting in abnormal promotion of tubulin polymerization [24]. Due to its high lipophilicity, aqueous solubilization of PTX is required [23]. As with most chemotherapeutic regimens, PTX causes significant side effects including peripheral neurotoxicity due to the lack of sufficient selectivity [25, 26]. Moreover, since the bio-accessibility of these anti-mitotic agents to tumors is limited, large doses are used. This consequently leads to untoward toxicity to healthy cells, as well as increased incidence of multidrug resistance (MDR) to multiple structurally and mechanistically distinct anticancer drugs [27].

Imidazoacridinones (IAs) are naturally fluorescent compounds having a hydrophobic weak base structure, characteristic to many anti-cancer chemotherapeutics. Certain acridinone derivatives are new potent cytotoxic agents which have shown promise against leukemia, melanoma, breast cancer and others [28, 29]. IAs apparently function via several mechanisms such as arresting the cell in the G_2_ phase of the cell cycle, inducing cell death, inhibiting cleavable complexes of topoisomerase II with DNA, intercalating into DNA and binding to the minor groove [29–31].

Conventional chemotherapy requires high drug doses, frequently causing severe toxic side effects due to insufficient selectivity, and exhibits limited efficacy due to the frequent emergence of MDR phenomena. One of the dominant mechanisms of MDR is mediated by efflux pumps of the ATP-Binding Cassette (ABC) superfamily which exert enhanced extrusion of various hydrophobic and amphiphilic cytotoxic drugs [28, 32–34]. One of the strategies to overcome MDR is by using novel NP systems [33]. Actively targeted NPs (e.g. based on HA targeting to its CD44 receptor) may evade drug efflux mechanisms by facilitating antitumor drug entry into tumor cells via receptor-mediated endocytosis, hence releasing the drug cargo from the endo-lysosomal compartment into the cytoplasm.

The aim of the current study was to devise a novel anticancer drug delivery system based on self-assembling Maillard HA-BSA conjugates, to characterize it, load it with an antitumor drug (such as PTX and the IA C-1375) and evaluate drug binding, selective uptake and cytotoxicity to cancer cells. The NPs were designed to selectively target CD44-overexpressing (e.g. ovarian) cancer cells, and HA served both as the hydrophilic block of the amphiphilic copolymer, and as the active targeting ligand.

## RESULTS AND DISCUSSION

### Conjugation of HA with BSA by the Maillard reaction

**Figure 1:**
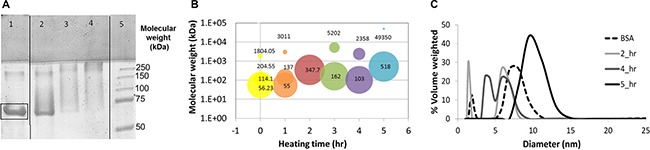
(**A**) SDS-PAGE of pure BSA (lane 1) and BSA-HA conjugate solutions at a 1:5 molar ratio of HA:BSA after 3, 7 and 21 hours (lane 2,3 and 4, respectively) of incubation at 60^°^C. Lane 5 depicts the standard MW markers. Molecular mass (**B**) and particle size distribution (c) in solutions of BSA-HA conjugates at a 1:5 molar ratio. [BSA-HA conjugates] = 1.2 mg protein/ml.

BSA-HA Maillard conjugates were prepared as described in the Methods section, and analyzed by SDS–PAGE. Figure 1A describes the SDS-PAGE analysis of BSA-HA conjugates at a molar ratio of 1:5 after different times of incubation at 60°C. HA has one reducing end group which may covalently react with an amine residue of lysine, arginine or tryptophan of BSA. Hence, BSA can readily form a Maillard reaction conjugate with a few molecules of HA, whereas an HA molecule can only react directly with a single BSA molecule. This characteristic is advantageous for the construction of small block copolymeric conjugates, rather than large network structures. Unheated BSA (lane 1) was predominantly unconjugated (∼67 kDa, marked by a rectangle), whereas upon increasing heating times, the primary 67 kDa BSA band gradually disappeared and some higher molecular mass bands emerged. This result provided direct evidence for the formation of higher MW BSA-HA Maillard conjugates. Notably, the samples remained white after heating, hence confirming that the Maillard reaction was successfully terminated at sufficiently early stages before melanoidins formed, but certainly after conjugation had occurred.

### Characterization of the self-assembled BSA-HA NPs in terms of size distribution and colloidal stability

The molecular mass and size distribution of the conjugate particles in solution were explored (Figure 1B and 1C, respectively). Figure 1B shows the molecular mass of BSA-HA conjugates at 5 heating times (each heating time is designated by a different color); the area of each circle at a given heating time indicates the percentage of each sub-population in the solution. It is evident that the most prevalent molecular mass species (56 kDa) in the mixture (of BSA and HA without heating) is relatively close to the molecular mass of BSA (∼67 kDa). HA alone (6–10 kDa; < 1 nm) was below the size-detection limit of the MALLS instrument used. After 1 hr of heating, the molecular mass had not significantly changed. However, additional heating time up to 5 hr at 60°C resulted in increased molecular mass and eventually in an almost mono-modal distribution. Expectedly, the molecular mass of BSA-HA conjugates increased with increasing Maillard reaction incubation times as well as with increasing HA:BSA molar ratio (Results not shown). We have chosen to continue our study with BSA-HA conjugates formed after 3 hr of Maillard reaction, having an average molecular mass of 162 kDa (Figure 1C). Such an average molecular mass of a conjugate may be composed of ∼13 HA molecules (average MW of 6.4 kDa) conjugated to a single BSA molecule (67 kDa). Nevertheless, it should be noted, that a disulfide-bonded BSA dimer with 4 conjugated HA molecules is also possible, as well as other combinations within the molecular mass dispersity of the products. BSA contains 59 lysine residues, (although only 30–35 are accessible for coupling reactions [35]), as well as 23 arginine-, 17 histidine- and 2 tryptophan residues [11, 12]. Hence, assuming 13 HA molecules and a single BSA molecule per conjugate, ∼13% of the Maillard-reactive amino acid side groups of BSA appear to have participated in the Maillard reaction. However, the moist heating involved in the Maillard reaction process may induce partial unfolding of SA, which should enhance the binding affinity to hydrophobic drugs, and self-assembly of the conjugates. On the other hand, one may raise the possibility that the potential unfolding might increase immunogenicity of SA. However, since SA is expected to be in the NP core, decorated with multiple HA molecules, this should markedly reduce the likelihood of immunogenicity.

Figure 1C exhibits the particle size distribution of BSA-HA at a 1:5 molar ratio as a function of heating time. Pure unheated BSA is also shown for comparison and it has two sub-populations: apparently monomers and aggregates of BSA. Pure HA (6–10 kDa; < 1 nm) is too small for analysis by dynamic light scattering (DLS), but the addition of HA to BSA, shifts the observed distribution to lower values, as may be inferred from the 2 hr graph. Larger sizes are expectedly observed with increased heating time. However, Figure 1C shows that at the various heating times, the BSA-HA conjugates are < 25 nm. One hr of heating resulted in three sub-populations: apparently, molecules of BSA alone and two sizes (5 and 10 nm) of BSA-HA particles, which may be BSA-HA conjugates, and their self-assembled aggregates. After 4 and 5 hr of heating, the size was found to be larger with a mono-modal distribution, suggesting that most of the BSA is HA-conjugated, and the conjugates were self-assembled into more uniform micellar aggregates. An interesting question was considered, whether or not the conjugates can self-assemble, as it is possible that when multiple HA molecules are bound per BSA molecule, a unimolecular “micelle” is formed with a hydrophobic core and a hydrophilic corona, which cannot self-assemble. Such a unimolecular micelle may still serve as a nanovehicle, but it is likely to be more difficult to load with significant drug cargo, and it may not be very effective in suppressing drug crystallization, as it would not be able to adsorb to hydrophobic drug nanocrystal surfaces.

**Figure 2:**
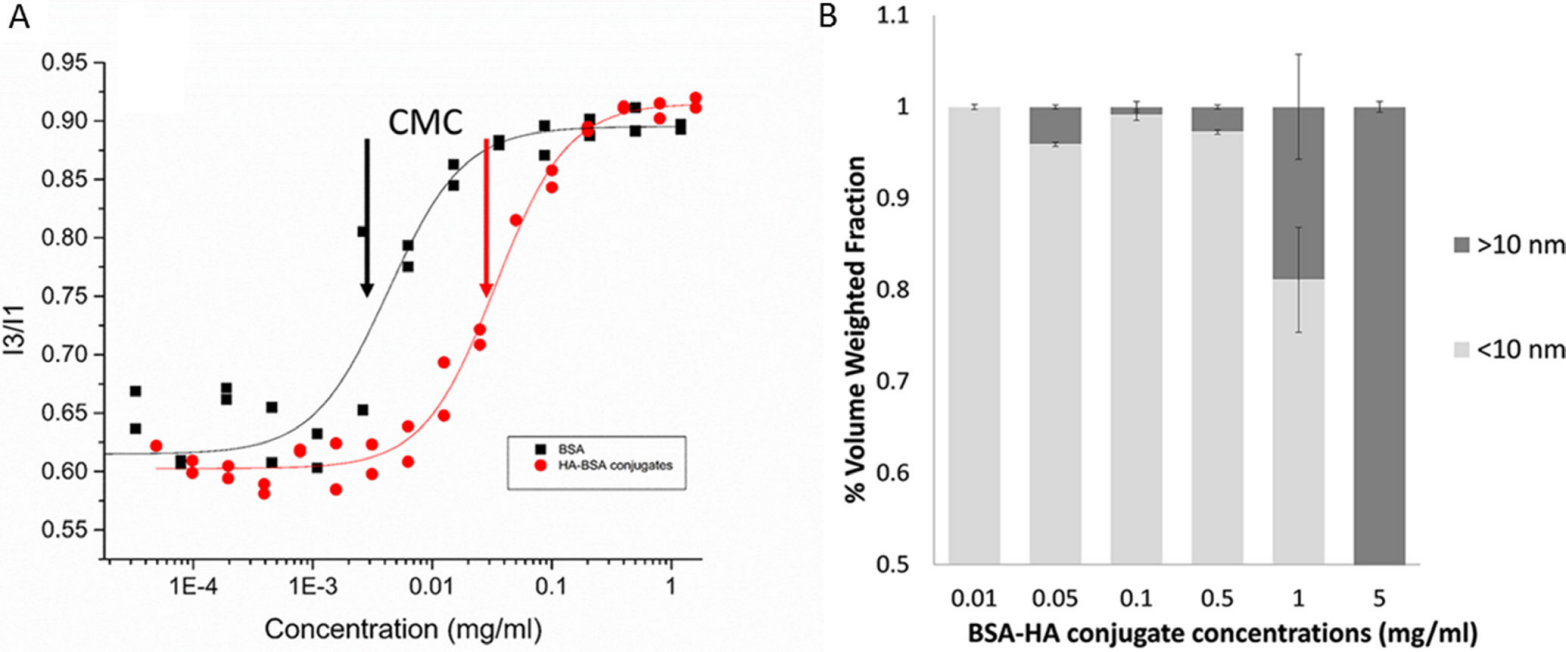
(**A**) Pyrene fluorescence I3:I1 ratio (386 and 374 nm, respectively) vs. BSA and BSA-HA conjugates concentration. [Pyrene] = 1.02 μM; excitation wavelength: 338 nm. The lines were obtained by model fitting based on Equations 1 and 2. Arrows represent the CMC: CMC (BSA) = (0.0041 ± 0.0008) mg/ml; CMC (HA-BSA conjugates) = (0.033 ± 0.002) mg/ml; (**B**) Volume weighted size distributions as a function of BSA-HA conjugates concentration.

To determine whether or not, conjugates prepared at different molar ratios and heating times can self-assemble, the micellization of BSA-HA conjugates was evaluated by determining their CMC, using pyrene as a fluorescent probe for hydrophobic domain formation [36] (Figure 2A). Pyrene is an uncharged hydrophobic molecule whose fluorescence is strongly influenced by the polarity of its nano-environment. The solubility of pyrene in water is very poor and its fluorescence is well characterized [37]. Figure 2A shows that the CMC of BSA-HA conjugates was expectedly higher compared to that of BSA alone [CMC (HA-BSA conjugates) = (0.033 ± 0.002) mg/ml; CMC (BSA) = (0.0041 ± 0.0008 mg/ml]. HA is a hydrophilic polymer, which interferes sterically and electrostatically with the hydrophobic interaction-driven aggregation or self-assembly of BSA molecules. Hence, the entire conjugate is more hydrophilic than pure BSA, and requires higher concentrations to induce its self-assembly. Figure 2B shows the size distributions of BSA-HA conjugates at different concentrations. At a concentration of 0.05 mg/ml, larger particles started to appear. This result is consistent with the CMC obtained using the pyrene fluorescence technique (∼0.033 mg/ml). Increasing BSA-HA conjugate concentrations >0.5 mg/ml resulted in larger NPs, whereas at a concentration of 5 mg/ml, all of the NPs were > 10 nm. Indeed, we found that the conjugates did self-assemble, and determined their CMC and micellization cooperativity. We observed that following different heating times and at all molar ratios studied (Results not shown), all BSA-HA conjugates self-assembled with a CMC value higher than that of unconjugated BSA (Figure 2A). These findings do not support the unimolecular micelle hypothesis, but favor instead, the self-assembling conjugates, which are preferable for higher drug loading.

**Figure 3:**
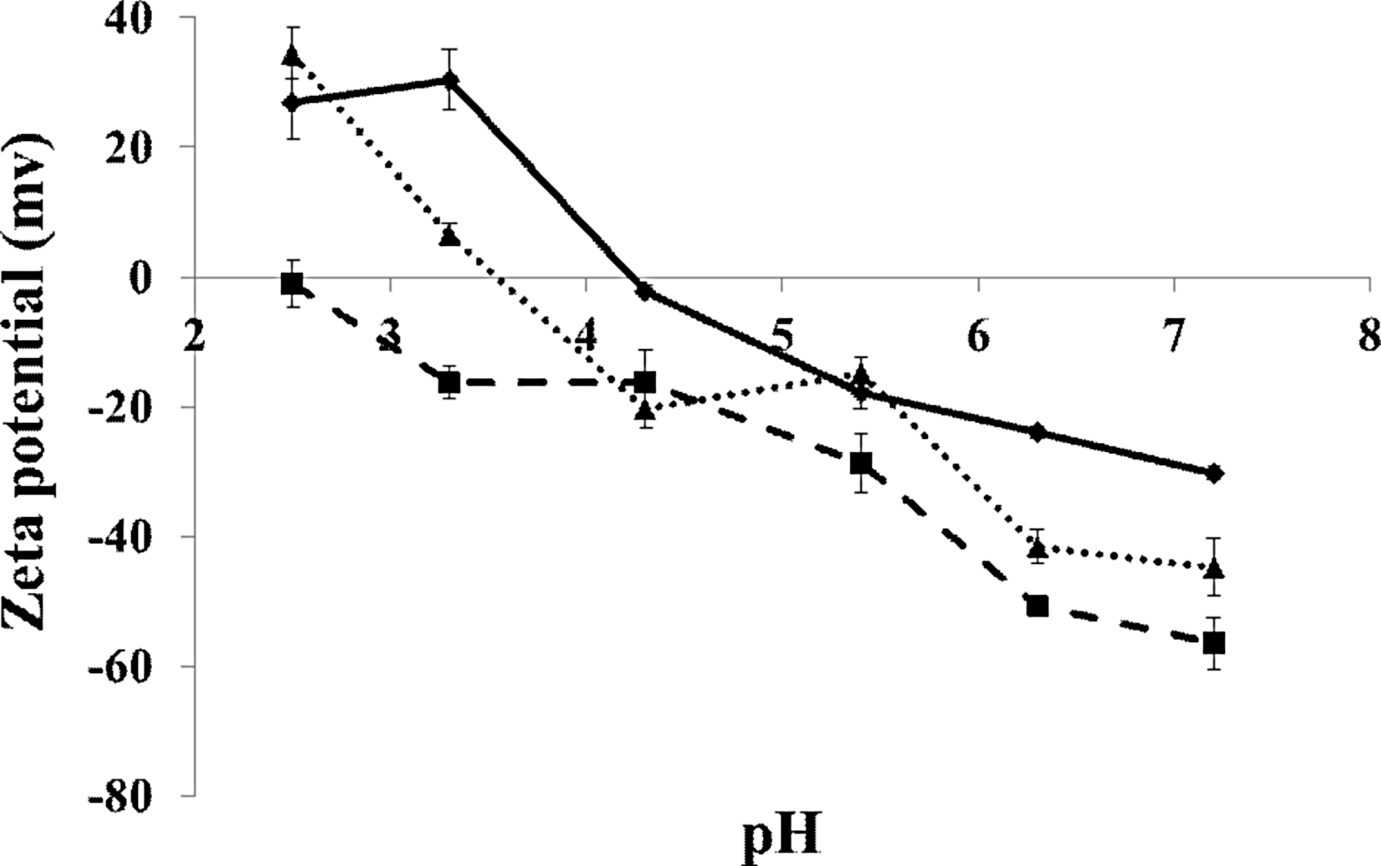
Zeta potential of BSA (diamonds), HA (squares) and BSA-HA conjugates (triangles) vs. pH. [BSA], [HA], [BSA-HA conjugates] = 0.6 mg protein/ml

An additional attribute studied herein was the zeta potential of BSA-HA conjugates, calculated based on the measured electrophoretic mobility. HA is a polyanion containing carboxylic groups, hence its negative charge increases with increasing pH. HA has a pKa value of ∼3.0 [38], thus zeta potential was negative, and it decreased when pH was increased in the measured range. Zeta potential measurements of pure BSA, pure HA and BSA-HA conjugate solutions in PBS *vs*. pH are depicted in Figure 3. The zeta potential of BSA was positive up to pH 4.3, in reasonable agreement with the published isoelectric point of BSA, 4.7 [39]. The zeta potential of BSA-HA conjugates was thus mostly found between those of pure BSA and pure HA. Suspensions with zeta potential values more negative than −40 mV usually have good colloidal stability [40, 41]. The zeta potential (Figure 3) can be calculated from the measured electrophoretic mobility using several available theoretical models. The choice of a suitable model is based on the value of κ·a where “a” is the particle size and “κ^−1^” is the Debye length. The two commonly used models are the Smoluchowski model, which is applicable when κ·a is significantly > 1 and the Huckel model, which is applicable when κ·a is < 1 [42]. In our current system, the product of κ·a was approximately 1.5, hence in this case, neither the Smoluchowski model nor the Huckel model were strictly applicable. However, the Smoluchowski model yields the lowest absolute value of zeta potential estimate for a given electrophoretic mobility value among the commonly used models [40; thus, stability prediction based on the Smoluchowski model is considered to be the most conservative. Therefore, taking the Smoluchowski model as a safe underestimation (in absolute value), the zeta potential of BSA-HA conjugates at pH 7.4 (the physiological pH which we mimicked using a PBS environment) was determined to be < −40 mV (ζ(Smoluchowski) ∼ −45 mV), indicating good colloidal stability.

### Entrapment of PTX within BSA-HA micellar NPs

### Interaction of PTX with BSA-HA conjugates

**Figure 4:**
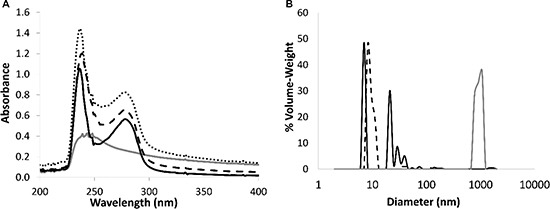
(**A**) Absorbance spectra of non-encapsulated PTX (20 μM, gray), BSA-HA conjugates at 1 mg protein/ml (solid black line), and of PTX in BSA-HA conjugates at 1 mg protein/ml (black, dashed line) compared to a mathematical summation (dotted line) of the absorbance spectra of non-encapsulated PTX and of the conjugate solution. DMSO concentration was 1.5 %. The molar ratio of PTX : BSA-HA conjugates was 2:1. (B) Particle size distribution determined using DLS, in solutions of BSA-HA conjugates (black, dashed line), non-encapsulated PTX (40 μM, gray) and of PTX-loaded BSA-HA NPs (solid black line) at the same concentrations of the respective components. The molar ratio of PTX:BSA-HA conjugates was 2:1.

The entrapment of PTX within the BSA-HA conjugate (5:1 molar ratio, 3 hr of heating) micelles was achieved by adding PTX (dissolved in DMSO) into a solution of the BSA-HA conjugates while continuously stirring. Figure 4A shows that the absorbance spectrum of PTX entrapped within BSA-HA conjugates was lower than the sum of the absorbance spectra of non-encapsulated PTX and of BSA-HA conjugates. This deviation from additivity evidences the binding of PTX to BSA-HA conjugates.

### Nanoparticle size distribution

Size distribution of the co-assembled BSA-HA-PTX NPs was analyzed by DLS (Figure 4B). The mean diameter of BSA-HA conjugates was < 15 nm. Non-encapsulated PTX exhibited a mean diameter of about 1000 nm (this is close to the upper detection limit of the DLS used). In fact, non-encapsulated PTX formed crystalline μm-sized aggregates. PTX-loaded BSA-HA conjugate NPs exhibited three particle size sub-populations: one of ∼7 nm, another of ∼20–40 nm and a small sub-population of ∼85 nm. The diameter of PTX-loaded BSA-HA conjugate NPs was similar to that of non-loaded BSA-HA conjugate NPs without the large sub-population typical to the free drug, demonstrating the effective entrapment of the drug by the BSA-HA conjugate NPs. Hence, these results indicate that BSA-HA conjugate NPs effectively stabilize PTX against aggregation and crystallization. The NP size distribution of the drug-loaded conjugates is readily compatible with endocytosis via binding to CD44, the HA receptor. Receptor-mediated endocytosis of NPs was consistently found to be optimal at 25 nm, and possible at least up to ∼60 nm [43], whereas for the EPR effect, which is expected to precede cellular uptake of these NPs, a range of 10–200 nm was reported to be suitable [3, 44].

### Cryo-TEM images

**Figure 5:**
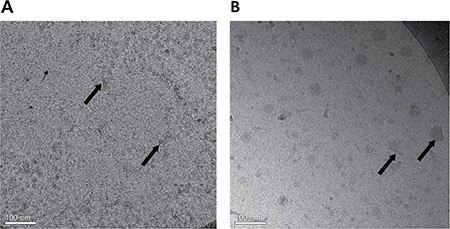
Cryo-TEM images of (**A**) BSA-HA conjugates (40 μM) without PTX. The grainy surface indicates BSA-HA conjugates (small arrows) and larger gray stains indicate self-assembled aggregates of BSA-HA conjugates (large arrows). (**B**) 2:1 molar ratio of PTX (80 μM) with BSA-HA conjugates; All samples contained 0.8% DMSO in PBS. The molar ratio of PTX and BSA-HA conjugates was 2:1. PTX aggregates and nanocrystals were coated with BSA-HA conjugates adsorbed to their surfaces (arrows in Figure 5B).

A closer observation, at a much higher magnification, was performed using Cryo-TEM, as shown in Figure 5A and 5B. BSA-HA conjugates were mostly <10 nm (based on the DLS- Figure 4B, dashed line, and Figure 5A, small arrows), apparently containing one or a few molecules of BSA, each decorated with a few molecules of HA (small covalently conjugated oligomers of BSA formed via disulfide bonding during the heating to form the Maillard conjugates), and self-assembled particles, probably containing many molecules of BSA-HA conjugates (10–20 nm, Figure 4B, dashed line, and Figure 5A, large arrows). In samples with added PTX (Figure 5B), small rod-like drug nanocrystals and spherical droplet-like drug aggregates were observed, surrounded by adsorbed BSA-HA conjugates (∼60–80 nm based on DLS Figure 4B, solid line, and Figure 5B), which stabilized them and prevented them from undergoing further crystal growth or aggregation. The particles were mostly between 10–60 nm (Figure 5B). Consistently, we have previously observed a similar effect of β-casein on PTX crystallization [45, 46].

### Entrapment of the imidazoacridinone C-1375 within the BSA-HA micellar NPs

### Interaction of IA C-1375 with BSA-HA conjugates

**Figure 6:**
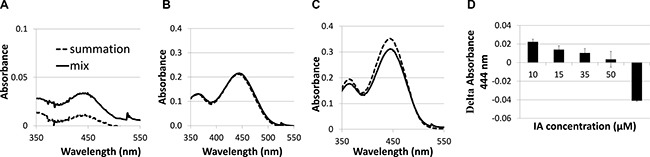
Absorbance spectra of imidazoacridinone C-1375 (**A**) 10 μM, (**B**) 50 μM, (**C**) 65 μM in BSA-HA conjugate (mix) at 1 mg protein/ml (solid black line) compared to a mathematical summation (dotted line) of the absorbance spectra of pure C-1375 and pure conjugate solutions at the same concentrations as in the mix; (**D**) Delta Absorbance (mix –sum) at 444 nm at different C-1375 concentrations.

UV-vis absorbance spectra were used to study the binding of IA C-1375 to BSA-HA conjugates. The samples were at a concentration of 1 mg/ml on a protein basis. Measurements were performed in duplicates at room temperature. Results are shown in Figure 6:

The mixed solution absorbance is expected to be additive, unless an association occurs between the observed solution components. It is evident that the mix absorbance curve is higher than the curve of the mathematical summation up to ∼50 μM C-1375 (Figure 6A). At 50 μM, the mathematical summation is nearly equal to the mix absorbance. These results are due to C-1375 sensitivity to its environment, as the absorbance of C-1375 changes in response to the hydrophobicity of its environment. We suggest that several BSA-HA conjugates interact through hydrophobic interactions of hydrophobic BSA domains, thereby creating a hydrophobic core in which the C-1375 is entrapped. At 65 μM of C-1375, the mathematical summation of the absorbance exceeds the absorbance of the mix. Above 50 μM of C-1375, the self-assembled conjugates apparently cannot entrap the excess ligand and the free C-1375 aggregates enhance its absorbance, thus the absorbance summation is higher than the absorbance of the mix.

**Figure 7:**
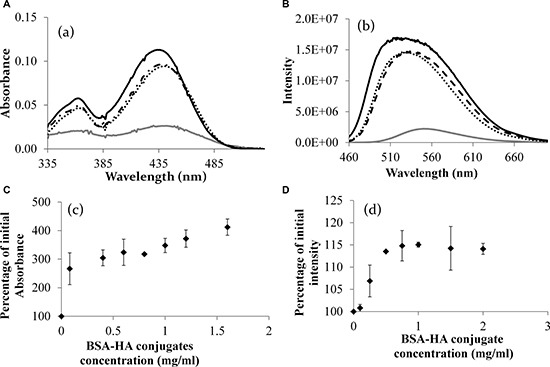
(**A**) The absorbance spectrum of C-1375 in different solvents. (**B**) Fluorescence intensity of pure C-1375 in different solvents (excitation: 435 nm). Methanol (solid black line), ethanol (dotted line), acetonitrile (dashed line), and water (gray line). (**C**) The percentage of the initial absorbance at 444 nm of pure C-1375 as a function of the concentration of the BSA-HA conjugate. (**D**) The percentage of the initial intensity of pure C-1375 vs. the concentration of the BSA-HA conjugate. [C-1375] = 15 μM.

Figure 6B presents the quantitative difference between the absorbance of the mix and the absorbance summation [47] of separate HA and BSA solutions. One can infer from Figure 6B that the maximal molar loading ratio of C-1375 in the BSA-HA conjugates is 8:1. Figure 7A displays the absorbance of C-1375 in water and in several organic solvents (ethanol, methanol, and acetonitrile). Figure 7C describes the absorbance percentage with respect to that of pure C-1375 as a function of the concentration of BSA-HA conjugates. The fluorescence emission spectrum in several organic solvents (water, ethanol, methanol, and acetonitrile) is described in Figure 7B. The fluorescence emission intensity at 550 nm (excitation at 435 nm) as a function of the concentration of the BSA-HA conjugates is presented in Figure 7D.

It is apparent from Figure 7A that C-1375 absorbance changes in response to the hydrophobicity of its environment. The absorbance of C-1375 in a hydrophilic solvent, water, is much lower compared to that in organic solvents. These results can help explaining the results shown in Figure 7C, in which increased BSA-HA concentration resulted in increased absorbance of C-1375 at 444 nm. We conclude that BSA-HA conjugates self-assembled and thus formed hydrophobic domains in which C-1375 was entrapped.

A hypochromic effect (blue shift) due to an increase in the hydrophobicity of the organic solvent resulting in a lower wavelength peak has been observed in Figure 7B. Furthermore, it is evident from Figure 7D that addition of BSA-HA conjugates to pure C-1375 increases the emission intensity of C-1375 at 550 nm. Therefore, both the increasing absorbance (Figure 7C) and the increasing fluorescence intensity (Figure 7D) of C-1375 with increasing BSA-HA conjugate concentrations, indicate the entrapment of this drug within the self-assembled BSA-HA conjugates.

### Nanoparticle size distribution

**Figure 8:**
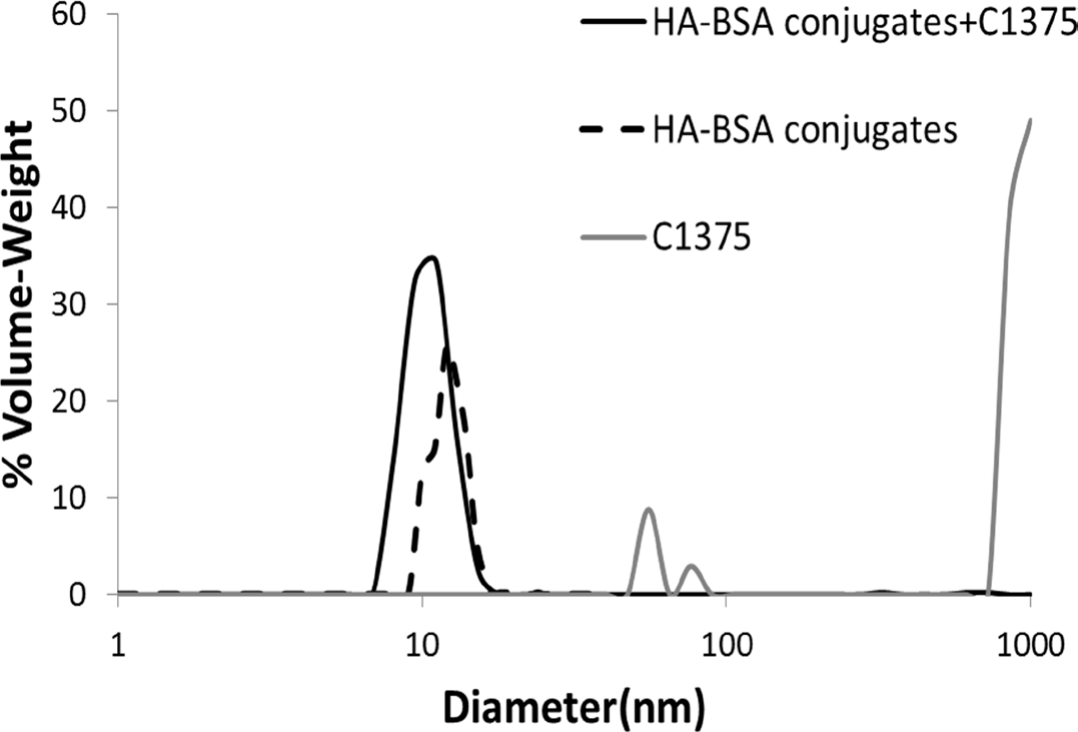
Particle size distribution determined using DLS, in solutions of BSA-HA conjugates (black dashed line), non-encapsulated IA C-1375 (80 μM, gray) and of C-1375 loaded BSA-HA NPs (solid black line) at the same concentrations of the respective components The molar ratio of C-1375:BSA-HA conjugates was 2:1.

Size distribution analysis of the co-assembled BSA-HA-C-1375 NPs was performed by DLS (Figure 8).

**Figure 9:**
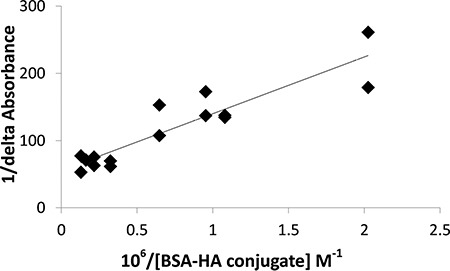
Benesi-Hildebrand plot (1/delta absorbance as a function of 10^6^/BSA-HA molar concentration, Eq. 3) to determine the C-1375 - BSA-HA conjugates association constant

For C-1375 alone, we observed three sub-populations, most of the particles were >1 μm, and two minor sub-populations were centered between 50 and 70 nm. However, for drug encapsulated in BSA-HA conjugates there was a single population, showing the same size distribution observed for BSA-HA conjugates alone, demonstrating the efficient entrapment of the drug by the BSA-HA conjugate NPs. To determine the association coefficient, we used the Benesi-Hildebrand model [47]. The absorbance was determined at 444 nm (at which C-1375 absorbs, while BSA-HA conjugates do not) as a function of BSA-HA concentration, thus the change in absorbance is caused only by the formation of complexes of C-1375 and BSA-HA conjugates; results are shown in Figure 9.

The results indicate that C-1375 has a high affinity for BSA-HA conjugates, and the association coefficient estimated by the Benesi-Hildebrand method was found to be:

Ka =(1.33 ± 0.26), 10^6^ M^−1^ i.e., a remarkably high association affinity.

### Selective targeting of BSA-HA NPs to CD44-expressing cancer cells

**Figure 10:**
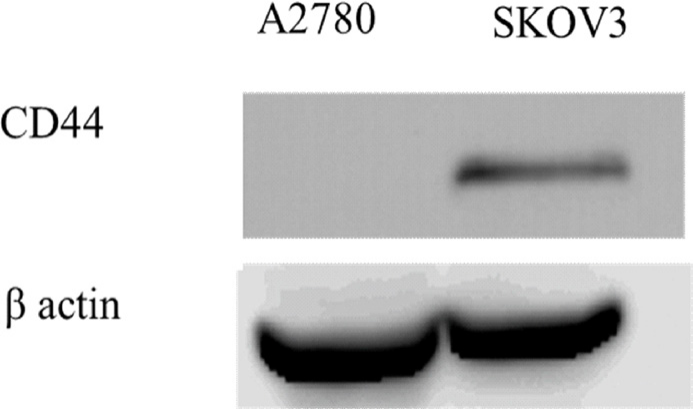
Western blot analysis of CD44 expression in A2780 and SKOV3 cells

Western blot analysis was used to quantify CD44 expression in two human ovarian carcinoma cell lines, A2780 and SKOV3 (Figure 10). This analysis confirmed CD44 overexpression (molecular mass ∼84 kDa) in SKOV3 cells, compared to A2780 cells, in which CD44 was not detectable.

The selective targeting of BSA-HA conjugates to human carcinoma cells overexpressing CD44 was studied using C-1375-loaded BSA-HA NPs. Three fluorescence channels were used during image capturing: (1) Blue for the viable DNA-dye Hoechst 33342; (2) Red for the viable lysosomal marker LysoTracker^®^ Red; and (3) Green for the naturally fluorescent cytotoxic agent C-1375. Co-localization of red and green fluorescent dyes (resulting in orange-yellow fluorescence) provided evidence for the internalization of C-1375-loaded BSA-HA NPs into endo-lysosomes, via CD44 receptor-mediated endocytosis. As detailed above, this selective targeting was explored using a cognate pair of two human ovarian cancer cell lines: SKOV3 which displays overexpression of CD44, the HA receptor, and A2780 cells which are devoid of CD44 expression.

**Figure 11:**
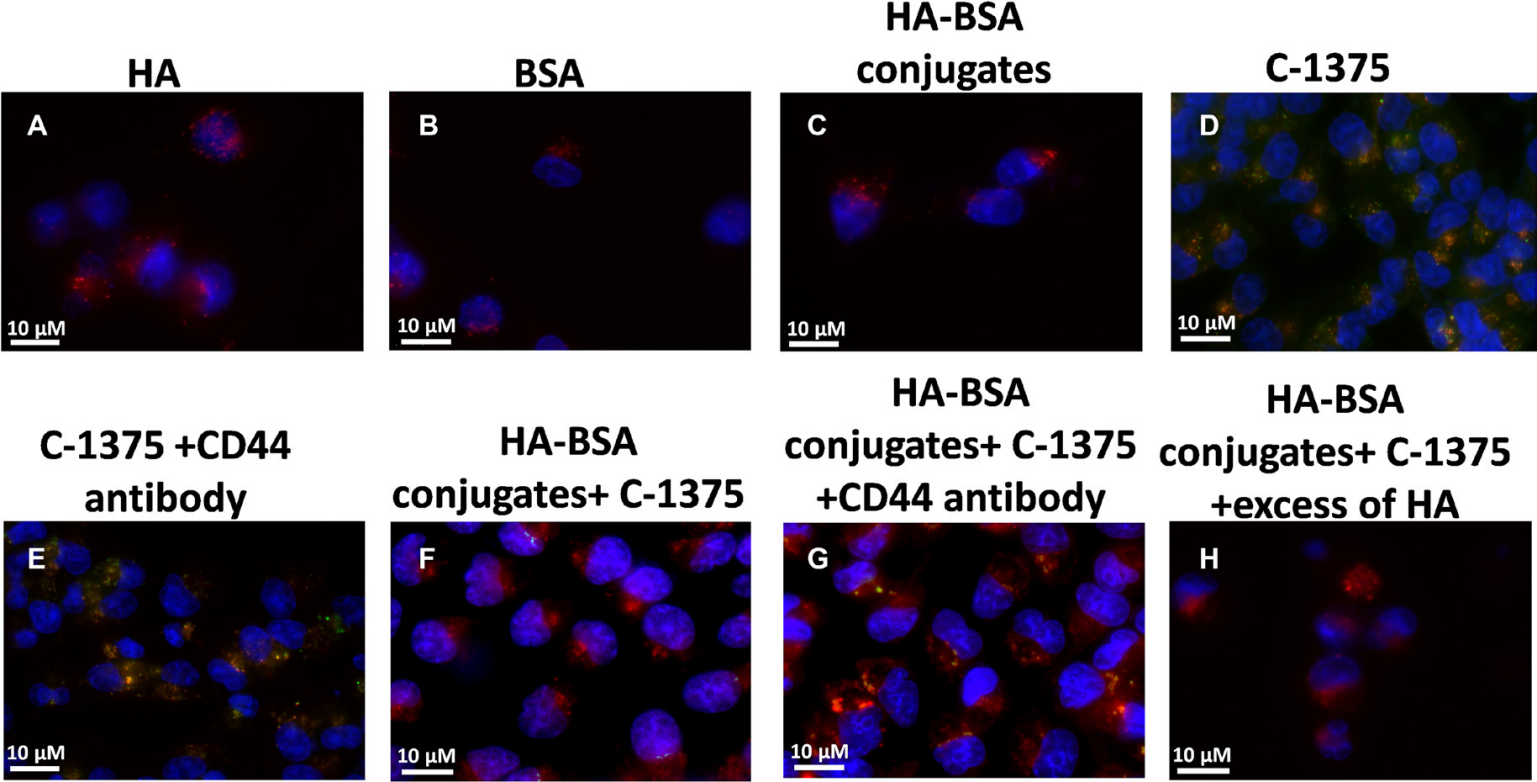
Ovarian carcinoma A2780 cells were incubated with the following samples: (**A**) HA; (**B**) BSA; (**C**) HA-BSA conjugates; (**D**) C-1375; (**E**) C-1375 with CD44-blocking antibody; (**F**) HA-BSA loaded with C-1375; (**G**) HA-BSA loaded with C-1375 + CD44 antibody; (**H**) HA-BSA loaded with C-1375 + free HA (molar ratio of free HA to conjugated HA was 100:1) for 1 hr at 37°C; BSA-HA NPs loaded with C-1375 (10 μM) were added for 2 hr prior to fluorescence cell imaging (anti-CD44 antibody at a final concentration of 10 μg/ml was added for 1 hr prior to BSA-HA conjugates). LysoTracker red DND99 (100 nM) was added for 1 hr prior to fluorescence cell imaging. The viable DNA dye Hoechst 33342 (2 mg/ml) was added immediately prior to microscopic analysis. Cells were then photographed using the Cell-Observer microscope at ×630 magnification.

**Figure 12:**
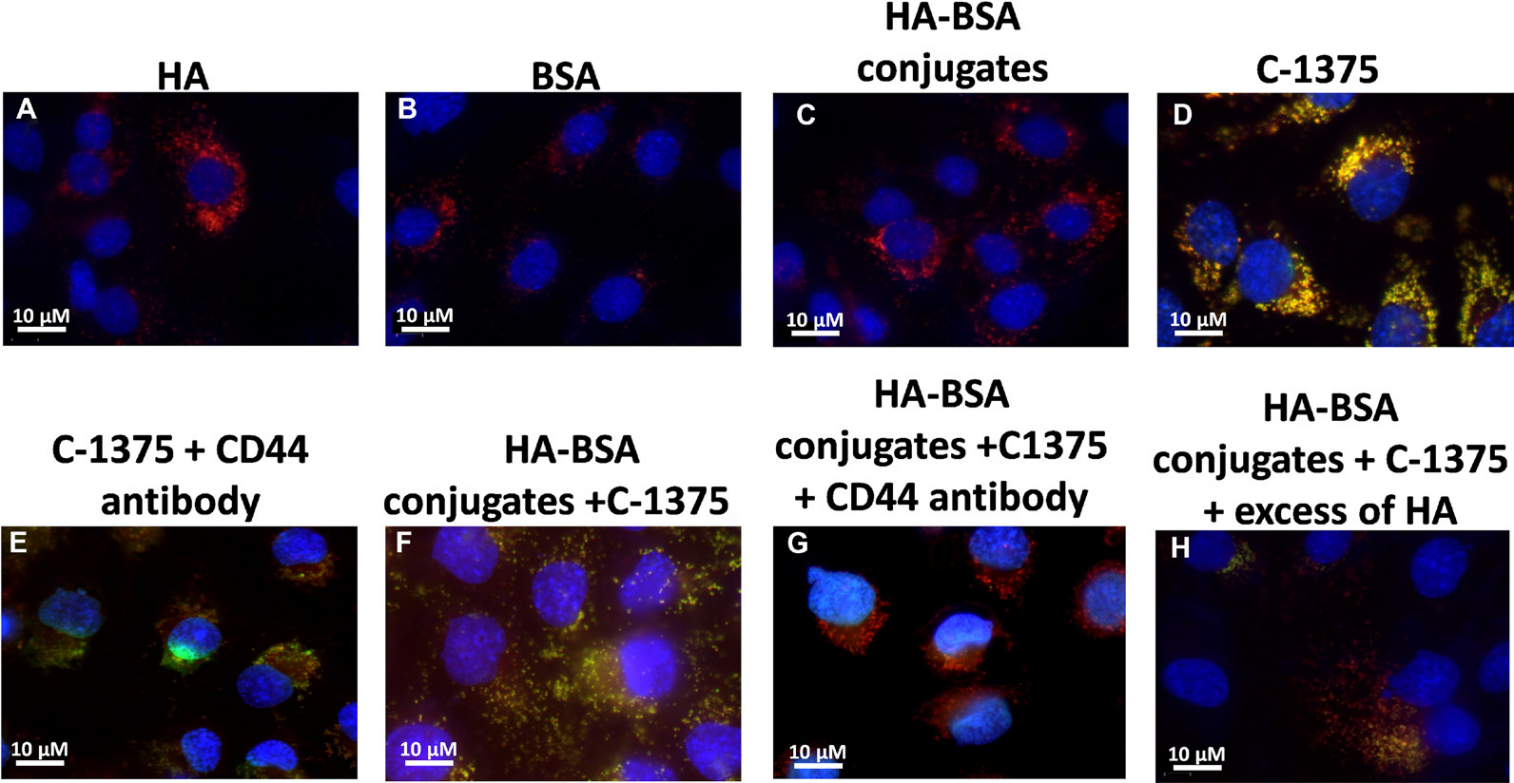
Ovarian carcinoma SKOV3 cells were incubated with the following samples: (**A**) HA; (**B**) BSA; (**C**) HA-BSA conjugates; (**D**) C-1375; (**E**) C-1375 with blocking antibody against CD44; (**F**) HA-BSA loaded with C-1375; (**G**) HA-BSA loaded with C-1375 + CD44 antibody; and (**H**) HA-BSA loaded with C-1375 + free HA (molar ratio of free HA to conjugated HA was 100:1) for 1 h at 37°C. BSA-HA NPs loaded with C-1375 (10 μM) were added for 2 hr prior to fluorescence cell imaging (CD44 antibody (10 μg/ml) was added for 1 hr prior to BSA-HA conjugates). LysoTracker red DND99 (100 nM) was added for 1 hr prior to fluorescence cell imaging. The viable DNA dye Hoechst 33342 (2 mg/ml) was added immediately prior to microscopic analysis. Cells were then photographed using the Cell-Observer microscope at ×630 magnification.

Expectedly, in the control groups, incubation of BSA, HA, and HA-BSA conjugates without C-1375 showed no green fluorescence (Figure 11A–11C and Figure 12A–12C). In contrast, when cells were incubated with the free C-1375 drug which is intrinsically green fluorescent (Figures 11D, 12D), it equally entered both SKOV-3 and A2780 cells by diffusion, independently of the presence of CD44. Furthermore, when cells were incubated with a drug that was encapsulated in the BSA-HA conjugates, the drug was delivered solely into CD44-containing SKOV3 cells (Figure 12F). These BSA-HA conjugates loaded with C-1375 were selectively taken up by human ovarian cancer SKOV3 cells overexpressing the HA receptor CD44 via endocytosis, but not by A2780 cells which are devoid of CD44 expression (Figure 11F). Following endocytosis, the drug-loaded NPs were found to concentrate within lysosomes, suggesting an efficient targeted uptake. This finding supports the conclusion that the uptake of the BSA-HA NPs was mediated by the CD44 receptor. To verify that this internalization of HA-decorated BSA NPs occurred via the HA receptor CD44, a 100-fold molar excess of free HA was added to SKOV3 cells (Figure 12H); this markedly eliminated the orange fluorescence signal, which reflects the co-localization of the green C-1375 fluorescence and the LysoTracker red fluorescence in lysosomes. This elimination of the orange fluorescence was due to a competition of free HA with HA-BSA conjugates for CD44 receptor binding. This competition did not occur in A2780 cells which are devoid of detectable CD44 levels (Figure 11H). To ensure that the selective internalization of HA-BSA NP was mediated via the CD44 receptor, we blocked CD44 activity using a CD44-blocking antibody. Blocking CD44 receptor activity markedly eliminated the orange fluorescence signal in human ovarian cancer SKOV3 cells overexpressing the HA receptor CD44, but not in A2780 cells which are devoid of CD44 expression (Figure 11G and Figure 12G). The fact that the Blocking occurred only in SKOV3 cells and not in A2780 cells which are devoid of detectable CD44 levels, demonstrated that HA-BSA NP conjugates were selectively internalized via endocytosis by ovarian cancer cells, which overexpress the HA receptor CD44, but not by cognate ovarian cancer cells lacking CD44. Hence, selective targeting was achieved with HA decoration of drug-loaded BSA NPs.

### Cytotoxicity assays

PTX displays a wide spectrum of antitumor activity, particularly against ovarian cancer, breast cancer, non-small cell lung cancer, head and neck tumors, Kaposi's sarcoma, and urologic malignancies. PTX is a highly lipophilic compound with a log *P value* of 3.96 and a very poor aqueous solubility of < 0.01 mg/ml [48]. Moreover, PTX is devoid of functional ionizable groups. Hence, the delivery of PTX to cancer cells is associated with substantial challenges. Until the introduction of Abraxane (a SA-bound PTX, currently used as the first line of treatment of metastatic pancreatic adenocarcinoma), the only commercial PTX formulation was a solution of PTX in Cremophor EL, which caused severe side effects. However, in recent years, a number of approaches have been reported to solubilize PTX using co-solvents and inclusion complexes. In addition, innovative approaches have been reported for passive targeting of tumors using NPs, nano-suspensions, liposomes, emulsions, micelles, implants, pastes and gels [49]. In our present study, the co-assembly of PTX with BSA-HA conjugates was achieved by adding PTX dissolved in DMSO, into a BSA-HA conjugate solution while continuously stirring. BSA-HA conjugates co-assembled with PTX most likely via hydrophobic interactions. Because PTX is highly hydrophobic, it tends to minimize water contact by aggregating and by adsorbing to hydrophobic domains of the BSA protein within the co-assembled NPs. The cytotoxic activity of non-encapsulated PTX was explored compared to PTX encapsulated in BSA-HA conjugates. Moreover, PTX encapsulated in BSA-HA conjugates was studied with excess free HA, as a competitor of the BSA-HA conjugates, for binding to the CD44 receptor.

**Figure 13:**
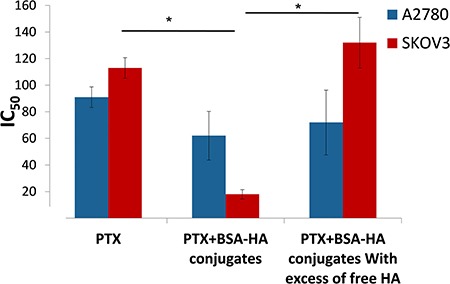
IC_50_ of PTX and that of PTX encapsulated within BSA-HA conjugates, for SKOV3 cells and A2780 cells with or without the addition of excess free HA Molar ratio of free HA to conjugated HA was 100:1; * denotes a *P-value* lower than 0.05.

The IC_50_ values of the four independent determinations are depicted in Figure 13. Free PTX was markedly less cytotoxic than PTX encapsulated in BSA-HA NPs, towards SKOV3 cells which overexpress CD44 (IC_50_: 113.9 ± 8.0 and 18.7 ± 3.5 respectively, *p-value* = 0.04), suggesting that lower drug concentrations would be needed in the presence of the conjugate NPs, which should significantly reduce harmful side effects.

**Figure 14:**
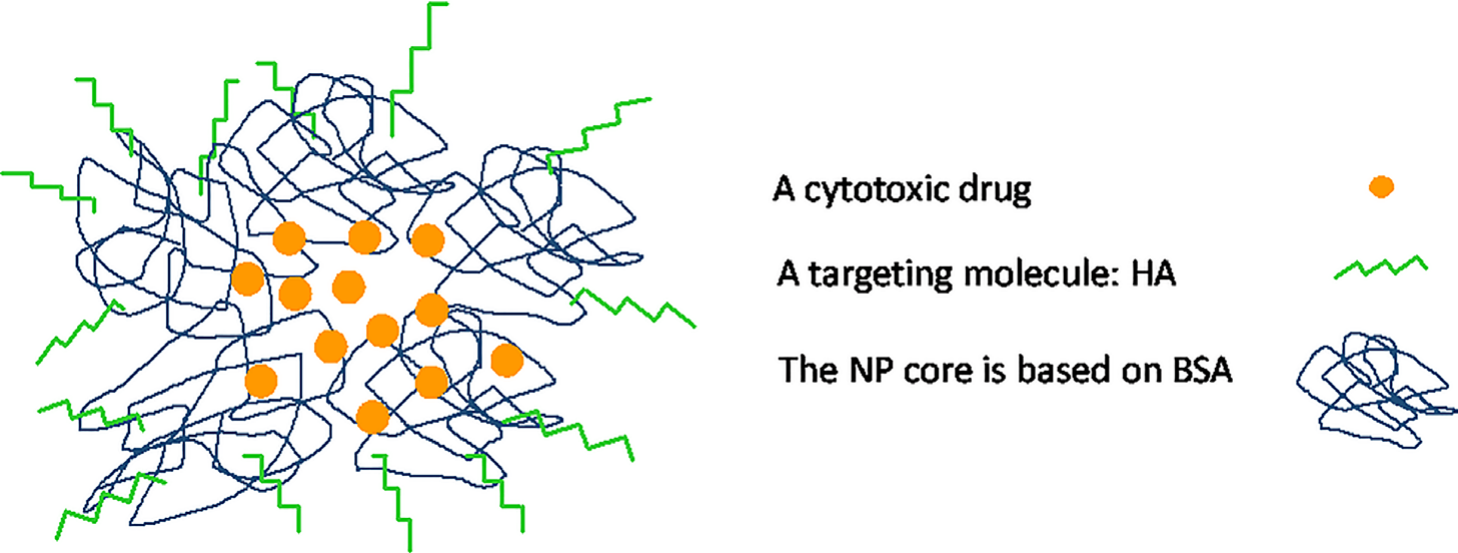
Schematic summary model of BSA-HA targeted nanovehicle entrapping a hydrophobic cytotoxic drug

The addition of free HA to SKOV3 cells markedly decreased the cytotoxicity of PTX-loaded BSA-HA NPs (IC_50_: 132.4 ± 17.4). This result is due to a competition of free HA with BSA-HA conjugates for binding to the CD44 receptor, thereby establishing a specific interaction of the HA-decorated NP-containing PTX with CD44. The IC_50_ value (Figure 13) of PTX entrapped in the BSA-HA conjugates in the presence of excess free HA was significantly higher than that in the absence of free HA, providing direct evidence for the CD44-mediated uptake of the drug-loaded NPs. Moreover, the cytotoxicity of free PTX and PTX-encapsulated in BSA-HA NPs was similar towards A2780 cells which lack the CD44 receptor (IC_50_: 91.9 ± 7.8 and 62.3 ± 18.3, respectively, *p-value* = 0.6). Incubation of A2780 ovarian cancer cells in the presence of excess free HA did not change the cytotoxicity of PTX-loaded BSA-HA NPs. These results support the conclusion that BSA-HA NP conjugates are selectively internalized by human ovarian cancer cells which overexpress the CD44 receptor but not by cells lacking this HA receptor. Based on these encouraging findings we propose a summary model of the BSA-HA nanovehicle entrapping cytotoxic drugs (e.g. PTX and C-1375, Fig 14).

In conclusion, our findings demonstrate that HA was covalently attached to BSA via the Maillard reaction by controlled heating. The mean diameter of BSA-HA NPs was <15 nm. BSA-HA conjugates self-assembled with a CMC value higher than that of BSA alone. BSA-HA conjugates stabilized PTX against aggregation and suppressed the growth of its nanocrystals, hence forming NPs of < 100 nm. C-1375 was found to be entrapped within a hydrophobic core formed by self-assembly of HA-BSA conjugates, exhibiting a remarkable binding constant (Ka) of (1.33 ± 0.26) × 10^6^ M^−1^. BSA-HA conjugates were selectively internalized by human ovarian cancer SKOV3 cells overexpressing the HA receptor, CD44, but not by cognate cells (A2780) lacking CD44 expression. PTX-loaded BSA-HA conjugate NPs displayed highly selective cytotoxicity to tumor cells overexpressing CD44 (SKOV3), but not to cells lacking CD44 (A2780); this difference could be markedly diminished by competition with excess free HA or by blocking CD44 with an antibody, hence evidencing in three ways the selective CD44-mediated uptake of these NPs. Based on these findings we conclude that BSA-HA conjugate NPs selectively targeted and entered CD44-overexpressing ovarian cancer cells via receptor-mediated endocytosis.

Taken collectively, these findings suggest that these BSA-HA conjugate NPs may be used as a nano-delivery platform for targeted delivery of hydrophobic chemotherapeutic drugs, and possibly also drug combinations including MDR inhibitors.

## MATERIALS AND METHODS

### Materials

Sodium hyaluronan with average MW of 6.4 kDa (∼34 monosaccharides) was purchased from Lifecore Biomedical, LLC (MN, USA). BSA and Hoechst 33342 were purchased from Sigma-Aldrich Ltd (Rehovot, Israel). PTX was purchased from Sigma-Aldrich and dissolved in DMSO (Loba Chemie, Mumbai, India) at a concentration of 10 mM. Rabbit monoclonal antibody (EPR1013Y) against human CD44 was from Abcam (Cambridge UK). Alexa Fluor^®^ 700 anti-mouse/human CD44 was purchased from ENCO (Petach-Tiqva Israel). Human ovarian adenocarcinoma cell lines SKOV3 and A2780 were cultured in McCoy's 5A and RPMI-1640 media, respectively (Biological Industries, Kibbutz Beit-Haemek, Israel), which were supplemented with 10% fetal bovine serum, 100 μg/ml penicillin, 100 units/ml streptomycin, 1% sodium pyruvate, 2.5% Hepes buffer to maintain physiological pH of 7.3 (final Hepes concentration of 25 mM) and 1% L-glutamine (Biological Industries). Cells were incubated at 37°C in a 5% CO_2_ humidified atmosphere. The imidazoacridinone C-1375 was synthesized by Prof. M. Cholody, B. Horowska and M. Konieczny and kindly provided by Prof. A. Skladanowski, Gdansk University, Gdansk, Poland.

### Methods

### Preparation of BSA-HA conjugates by the Maillard reaction

BSA-HA conjugates were prepared as follows: solutions of BSA and HA, at different molar ratios, were prepared in HPLC-grade water. To facilitate the Maillard reaction, the pH of the solutions was adjusted to 8 by adding NaOH. The solutions were then freeze-dried and placed over a saturated KBr solution in a desiccator, to maintain constant relative humidity of 79.9%. The desiccator was kept in a thermostatic oven at 60°C [50] for different heating times of 1 to 23 hr. Samples were then removed from the oven, freeze-dried and stored at −20°C for further analysis. The molecular mass of BSA-HA conjugates was evaluated by sodium dodecyl sulfate polyacrylamide gel electrophoresis (SDS–PAGE). Briefly, proteins were resolved on 10% polyacrylamide gels containing SDS as previously described [46].

### Molecular weight distribution

The molecular weight of BSA-HA conjugates (1:5 molar ratio) was determined in duplicates at room temperature, using size exclusion HPLC with a multi-angle laser light scattering (MALLS) detector (DAWN EOS, Wyatt Technology Corporation Santa Barbara, CA, USA) combined with a refractive index based concentration detector (Optilab Rex, Wyatt Technology). The BSA-HA conjugates sample concentration was 1.2 mg protein/ml.

### Particle size distribution

Particle size distribution was evaluated using a DLS analyzer (Nicomp^TM^ 380, PSS Inc., Santa Barbara, CA, USA). Measurements were performed in triplicates at room temperature.

### Zeta-potential measurements

Nicomp 380 ZLS analyzer was used for zeta potential measurements of BSA, HA and BSA-HA conjugates. Samples were dissolved at 20 mM Phosphate/phosphate-citrate buffers at pH values of 2.5–7.2 and a concentration of 0.6 mg protein/ml and stirred overnight.

### Determination of critical micellization concentration

The self-assembly of BSA-HA conjugates was studied by determining the critical micellization concentration (CMC) of the conjugates using pyrene as a hydrophobic fluorescent probe, which alters its emission spectrum in hydrophobic environments. The CMC was determined in the presence of a low and constant pyrene concentration (1 μM), based on the increase in pyrene fluorescence intensity ratio I3:I1 during its partitioning into hydrophobic domains formed upon micellization [36]. The CMC was estimated by using the following semi-empirical mathematical model (Equations 1,2) we have previously developed [51] to describe protein micellization:

$$\frac{I_{3}}{I_{1}}=\left(1-f\right)\frac{I_{3}}{I_{1}}{|}_{C=0}+f\frac{I_{3}}{I_{1}}{|}_{c=\infty }$$ (1)

$$f=\frac{1}{1+{\left(\frac{CMC}{C}\right)}^{K}}$$ (2)

Where C is the protein concentration, f is the fraction of pyrene entrapped in micelles, and K is the micellization cooperativity parameter. The self-assembly of BSA-HA conjugates was also studied by determining the diameter of the NPs at different concentrations.

### Entrapment of PTX within BSA-HA conjugates

Entrapment of PTX within BSA-HA conjugates was achieved by adding PTX dissolved in DMSO ( 1.5%), into a solution of the BSA-HA conjugates while continuously stirring, similarly to the method we previously used to entrap PTX and other drugs in β-casein micelles [52–54]. DMSO was not removed as it is not harmful to cells at such low concentrations [55], and is commonly used in IV chemotherapeutic treatments.

### Cryogenic-transmission electron microscopy (cryo-TEM) imaging

Cryo-TEM was used for the imaging of PTX entrapped in BSA-HA conjugate NPs at a 2:1 molar ratio in phosphate buffer containing 0.8% DMSO. An image of BSA-HA conjugates in the same buffer solution was taken for comparison. Vitrified specimens for cryo-TEM were prepared in a controlled environment vitrification system at 25 ± 0.01°C and 95–99% relative humidity, to ensure a constant temperature and to avoid water loss from the solution during sample preparation. The specimens were prepared as thin liquid films (10–500 nm thick), on perforated carbon films and quench-frozen in liquid ethane at its freezing point (−183°C). This procedure is designed to prevent water crystallization during thermal fixation. In this manner, component segregation and rearrangement are mostly prevented, and the original fluid nanostructure is preserved. The technique and apparatus were previously described in detail by Bellare et al., [56]. The vitrified samples were then stored under liquid nitrogen (-196°C), transferred to a Gatan 626 cooling holder via its “work station”, and observed in a Philips CM120 microscope at about 175°C. Images were recorded at a 120 kV acceleration voltage, in a low-dose mode, to minimize electron-beam radiation damage. Gatan Multi Scan 791 cooled CCD camera was used to acquire the images, with the Digital Micrograph 3.1 software package. Images were recorded under focus of about 2 μm to enhance phase contrast.

### Binding studies by UV-vis absorbance spectra

Ultraviolet-visible (UV-vis) absorbance spectroscopy was used to study the binding of drugs (PTX or C-1375) to BSA-HA conjugates. The samples were set at a concentration of 1 mg protein/ml. The differences between the absorbance spectra of the mixed BSA-HA-drug solution (PTX or C-1375) and the mathematical summation of the spectra of BSA-HA solution and ligand solution (PTX or C-1375) served to assess whether or not binding occurred. UV-Vis spectra were measured by a UV-Vis spectrophotometer (Ultrospec 3000, GE Healthcare).

### Binding constant determination

The binding constant was determined using the Benesi-Hildebrand method [57, 58] (Equation 3). The absorbance at 444 nm of C-1375 in phosphate buffer and different concentrations of BSA-HA conjugates was determined.

$$\frac{1}{\Delta A}=\frac{1}{K\text{·}\Delta \in_{444}[C-1375]}\text{·}\frac{1}{\left[BSA-HA\;conjugates\right]}+\frac{1}{\Delta \in_{444}[BSA-HA\;conjugates]}$$ (3)

ΔA is the change in absorbance at 444 nm at different concentrations of BSA-HA conjugates varying from 0 to 1.25 mg protein/ml. Δε 500 is the differential extinction coefficient at 444 nm. The final concentration of C-1375 was 15 μM. The extinction coefficient of C-1375 at 444 nm was determined according to Beer-Lambert law [59].

### Binding studies by spectrofluorometry

Fluorescence measurements were performed at room temperature, using a Fluorolog 3–22 spectrofluorometer (Horiba, Jobin Yvon, and Longjumeau, France). The fluorescence emission spectrum of C-1375 in several organic solvents (water, ethanol, methanol, and acetonitrile) was determined and compared to the fluorescence emission of C-1375 in the presence of increasing concentrations of BSA-HA conjugates.

### Tissue culture

Human ovarian adenocarcinoma cell lines SKOV-3 and A2780 were kindly provided by Dr. I. Chefetz-Menaker, the Lab of Prof. Gil Mor, Yale University, New Haven, Connecticut, USA. These tumor cell lines were cultured in McCoy's SKOV-3 and RPMI-1640 growth media (A2780), supplemented with 10% fetal calf serum and 1 mM L-glutamine. All cell lines were grown in a 5% CO_2_-humidified incubator at 37°C.

### Western blot analysis

To examine the expression of CD44 in the two human ovarian cancer cell lines [A2780 and SKOV3], total cell protein extract was isolated from 2 × 10^7^ cells in a lysis buffer containing 50 mM Tris at pH = 7.5, 50 mM β-mercaptoethanol, 0.5% Triton ×100, and 1 mM EGTA in DDW. Following 1 hr incubation on ice, the extract was centrifuged at 14,000xg for 30 min at 4°C and the supernatant was collected. Protein content was determined using the Bio-Rad protein assay. Protein aliquots (60 μg) were resolved by electrophoresis on 10% polyacrylamide gels containing SDS and electroblotted onto a Protran BA83-cellulose nitrate membrane (Whatman). The blots were then blocked for 1 hr at room temperature in Tris buffered saline (TBS) containing 150 mM NaCl, 20 mM Tris-base at pH 7.6 containing 1% skim milk. For the detection of CD44, rabbit monoclonal antibody (EPR1013Y) was used at a dilution of 1:5,000. Blots were then rinsed and reacted with a secondary antibody consisting of horseradish peroxidase (HRP)-conjugated anti-rabbit IgG (1:10,000 dilution, Invitrogen, Carlsbad, CA) for 1 hr at room temperature. Following three 10-min washes in TBS at room temperature, enhanced chemiluminescence detection was performed. To normalize for any differences in actual protein loading, the blots were stripped and re-probed with a monoclonal antibody to β-actin (1:4,000 dilution; Chemicon, Temecula, CA).

### Selective targeting of BSA-HA NPs to CD44 expressing cancer cells

The selective targeting of BSA-HA conjugates to human carcinoma cells which overexpress CD44 was studied using C-1375-loaded BSA-HA NPs. This selective targeting was explored using a pair of two human ovarian cancer cell lines: SKOV3 which displays overexpression of CD44, the HA receptor, and A2780 cells which are devoid of CD44 expression. Cells were seeded in culture dishes (35 mm) containing cover glass bottom (2×10^4^ cells/2 ml; World Precision Instruments, NBT New Biotechnology, Israel) and grown in medium (McCoy and RPMI-1640 for SKOV3 cells and A2780 cells, respectively). BSA-HA NPs loaded with C-1375 (10 μM) were added for 2 hr prior to fluorescence cell imaging (anti-CD44 antibody at a final concentration of 10 μg/ml was added for 1 hr prior to BSA-HA conjugates at the relevant samples). Cells were then washed with PBS, and LysoTracker red DND99 (100 nM) was added for 1 hr prior to fluorescence cell imaging. Prior to analysis, cells were suspended in PBS supplemented with 1 mM CaCl_2_, 1 mM MgCl_2_ and 10 mM D-glucose. Then, random colonies were analyzed using Zeiss inverted Cell-Observer microscope. The viable DNA dye Hoechst 33342 (2 mg/ml) was added immediately prior to microscopic analysis. Three fluorescence channels were used during image capturing: (1) Blue for the viable DNA-dye Hoechst 33342; (2) Red for the viable lysosomal marker LysoTracker® Red; and (3) Green for the naturally fluorescent cytotoxic agent C-1375. Co-localization of red and green fluorescent dyes (resulting in orange-yellow fluorescence) provided evidence for the internalization of C-1375-loaded BSA-HA NPs into endo-lysosomes, via CD44 receptor-mediated endocytosis.

### Cytotoxicity Assays

Cytotoxic activity was determined using an XTT-based colorimetric cell proliferation kit (Biological Industries Israel Beit-Haemek Ltd.). Exponentially growing cells were seeded at 1×10^4^ cells/well in 96-well plates. Following an overnight incubation, cells were exposed to increasing drug concentrations (10 μl drug solution/well) for 2 hr. Cellular viability was determined by adding the XTT reagent and an activation reagent at 50:1 v/v ratio and incubating for 1 hr at 37°C. Following solubilization of the formed formazan dye, absorbance was determined by a microplate reader. Values presented are means of three independent experiments, each performed in triplicates.

### Statistical analysis

The characterization of BSA-HA samples and the encapsulation of PTX were performed in duplicates for all experiments. An average value and a standard error were calculated at each point. The cytotoxicity assays were performed in triplicates. The statistical analysis of variance of the encapsulated (in the presence or absence of excess free HA) *vs*. the non-encapsulated PTX, was performed using a two-sample *T-test*, assuming equal variances, using Microsoft Excel™.
